# Use of Emergency Department Pharmacists in Emergency Medicine Resident Milestone Assessment

**DOI:** 10.5811/westjem.2018.10.37958

**Published:** 2018-12-05

**Authors:** Starr-Mar’ee C. Bedy, Kara B. Goddard, Julie A.W. Stilley, Christopher S. Sampson

**Affiliations:** University of Missouri-Columbia, Department of Emergency Medicine, Columbia, Missouri

## Abstract

**Introduction:**

The use of competency-based milestones for emergency medicine (EM) was mandated by the Accreditation Council for Graduate Medical Education in 2013. However, clinical competency committees (CCC) may lack diverse, objective data to assess these new competencies. To remedy the lack of objective data when assessing the pharmacotherapy sub-competency (PC5) we introduced a unique approach that actively involves departmental clinical pharmacists in determining the milestone level achieved by the resident.

**Methods:**

Our pharmacists assess the pharmacotherapy knowledge of the residents through multiple methods: direct observation of orders, communication with the residents while performing patient care within the emergency department (ED), and real-time chart review. This observation occurs informally on a daily basis in the ED and is incorporated into the routine work of the pharmacist. The pharmacists use the PC5 sub-competency as their standard evaluation tool in this setting to keep all assessments consistent.

**Results:**

Since our residency program introduced pharmacist assessment of resident pharmacotherapy knowledge, the CCC has conducted seven biannual meetings. Of the 120 separate PC5 sub-competency assessments made during those meetings there was 100% agreement between the pharmacist’s assessment and the CCC’s final assessment of the trainee. A survey of the CCC members concluded that the pharmacists’ assessments were useful and aided in accurate resident evaluation.

**Conclusion:**

The use of ED pharmacists in assessing the pharmacotherapy sub-competency provides important information used in resident assessment of the PC5 milestone.

## INTRODUCTION

The use of competency-based milestones for emergency medicine (EM) began in July 2013 as mandated by the Accreditation Council for Graduate Medical Education (ACGME).^1^ An ACGME milestone is an observable behavior of the EM resident that fits within five levels of proficiency. These are graded on a scale from one to five. A level 1 proficiency is expected of a medical school graduate, while a level 5 is the expected achievement only after years of practice in the specialty. A level 4 rating is expected of a graduating EM resident according to standards set by the American Board of Emergency Medicine.^3^ Since their introduction many residency programs have anecdotally noted difficulty in implementing some of these milestones as an assessment tool. As stated by Carter: “Milestones are complex, multifaceted, and sometimes fairly dense descriptions of a level of attainment on the road to competence… Milestones often are meant to be assessed by using multiple modalities.”^2^

Within the 23 EM milestones, pharmacotherapy (PC5) is a subset of the “patient care” competency. In assessing PC5 proficiency the observer ideally should document how the EM resident “[s]elects and prescribes appropriate pharmaceutical agents based upon relevant considerations such as mechanism of action, intended effect, financial considerations, possible adverse effects, patient preferences, allergies, potential drug-food and drug-drug interactions, institutional policies, and clinical guidelines; and effectively combines agents and monitors and intervenes in the advent of adverse effects in the emergency department” (ED).^4^ The suggested assessment methods for this sub-competency according to the ACGME include the following: Standardized Direct Observation Tool (SDOT), portfolio, simulation, oral boards, global ratings, and medical knowledge examinations.^3^ One difficulty in using these multiple modalities is the lack of objective, multi-source data available to the clinical competency committees (CCC) when assessing trainees. To increase the amount of objective data obtained for the pharmacotherapy sub-competency, our institution took a unique approach by actively involving pharmacists. We used assessments from ED-based pharmacists to provide 180° evaluations that can be an objective component of the level-assessment achieved by residents in the PC5 sub-competency.

## METHODS

Originally, the CCC for our ACGME-accredited residency program was comprised of five emergency physicians, the residency program director (PD), and the associate PD. At the start of our residency program, the two ED pharmacists jointly submitted their PC5 evaluations for each resident to the CCC. The departmental pharmacist assesses residents’ pharmacotherapy knowledge by direct observation of their verbal and electronic medical record orders, communication with them while performing patient care within the ED, and chart review of active ED patients. These observations occur on a daily basis and are incorporated into the pharmacists’ routine work without any significant, additional burden on them.

The pharmacists created an Excel spreadsheet to document their observations. The documentation was based on clinical scenarios that matched the specific medication knowledge evaluated for the resident’s postgraduate year (PGY). Then, the pharmacists met to synthesize their comments and jointly assign a numeric value for each resident’s milestone assessment. The pharmacists used the PC5 sub-competency as their standard assessment tool to keep all assessments consistent. To provide more assessment opportunities, the pharmacists also observed resident performance during yearly mock oral boards via real-time video. Mock oral boards included two single-case encounters and one triple-case encounter. Encounters were the same for all levels of residents, and the number of pharmacotherapy choices for an encounter varied significantly depending on the type and underlying diagnoses.

Since PC5 includes evaluating patient allergies, every case had at least one pharmacotherapy evaluation point. One pharmacist reviewed each case and documented the pharmacotherapy decision-making. Results were then discussed between the ED pharmacists to determine the overall score for the performance. Observation of the same case repeatedly with different residents likely increased pharmacist evaluation reliability. During these sessions, the pharmacists only assessed the medication management sections of the cases. A separate tool was created for use during the mock oral boards (Table 1). The pharmacists use the results of this tool to help incorporate their final resident evaluation on PC5.

The pharmacists also assessed the residents’ specific medication knowledge. Based on consensus between the residency leadership and the pharmacists, medication knowledge was separated into basic, intermediate and advanced skill levels, which were applied to each resident based on his/her level of training. Our current assessment plan is as follows:

- PGY-1 – analgesia, antiemetics, and antihypertensives
- PGY 2 – anti-infectives, pediatric medication dosing, procedural sedation medications
- PGY 3 – cardiorespiratory arrest medications, vasopressors, anti-coagulant reversal agents.

PGY-3 assessments include more advanced medications. For example, we placed cardiorespiratory arrest medications in this category because the more senior residents serve in the team leader role and lead in the resuscitation of the critically ill patient.

Our EM residency program is based at an urban, academic center with annual ED volume of 55,000 patients at our main hospital. The program started in 2014 as a three-year program with eight residents per class, with an increase to 10 per class in 2018. This quality improvement project was submitted to the institutional review board (IRB) and was exempt from formal IRB approval requirements.

Our ED is staffed by two full-time pharmacists who completed Doctor of Pharmacy degrees (PharmD) at United States universities, followed by two years of residency training in pharmacy practice and EM. Both were board-certified pharmacotherapy specialists in 2012. They were qualified as preceptors by the American Society of Health-System Pharmacists residency accreditation standards with experience evaluating pharmacy students and residents against educational standards, and are active members of the pharmacy department’s Residency Advisory Committee. Program-specific evaluation education was provided at two points in the process. The first occurred when the pharmacists were asked to submit evaluations to the CCC and the PD explained to them how the PC5 sub-competency was evaluated. The second occurred when the pharmacists were asked to join the CCC. At that point the PD provided additional information on the evaluation of non-pharmacotherapy milestones and reviewed all of the other requirements for EM residency graduation.

The pharmacists are present in the ED 10 hours a day/seven days a week, including most holidays, from noon to 10 pm. Their workspace is centrally located in the physician and nursing area in the center of the ED. This location makes them accessible to staff and enables them to assess our residents in real time. The residents work eight- to 12-hour shifts; therefore, the shift variability between the pharmacists and residents enables them to encounter two separate shifts of EM residents. Typically, three EM residents are working in the ED at one time: one PGY-1, one PGY-2, and one PGY-3 per shift. Based on historical data, the PGY-1 sees 1.2 patients per hour on average, with the PGY-2 and PGY-3 seeing approximately 1.5 patients per hour each. Given the pharmacist’s 10-hour shift, this translates to the pharmacist seeing approximately 4.2 resident patients per EM resident per hour for the three residents. This does not include the patients of residents rotating from off service, physician assistants, nurse practitioners, medical students, and attending-only patients for whom the pharmacists also provide care and review charts. Therefore, on any given day the approximate 42 patients seen per shift by the pharmacists to evaluate the EM residents does not add to their normal workflow.

The pharmacist assists at the bedside with critically ill patients, procedural sedations, and overdoses, and performs chart review on other patients. Due to the close proximity of pharmacist and physician workstations, the pharmacist is able to continually evaluate clinical performance throughout his/her shift while the EM residents care for patients of all acuities. The pharmacist conducts chart review of the residents’ medication orders, observes their bedside care, and listens to them as they present their plans to the attendings. For patients not receiving any medication therapy, the pharmacist review of the EM resident is minimal; nevertheless, the pharmacist’s observations could add to other areas of resident evaluation (e.g., professional values and communication skills).

As stated above, the daily observation of residents does not add to our ED pharmacists’ workload. Their only additional duties are to attend the mock oral board sessions and prepare an assessment report for the CCC. The CCC meets informally monthly in order to address resident education concerns early. The CCC performs formal resident assessments biannually. During the biannual CCC meeting, a report is generated by the pharmacists for each resident being assessed. Using the historical data of average patients per hour and pharmacist shift length, the average EM resident had approximately 50 patient encounters reviewed, which made up the semi-annual report prepared by the ED pharmacists.

**Figure 1:**
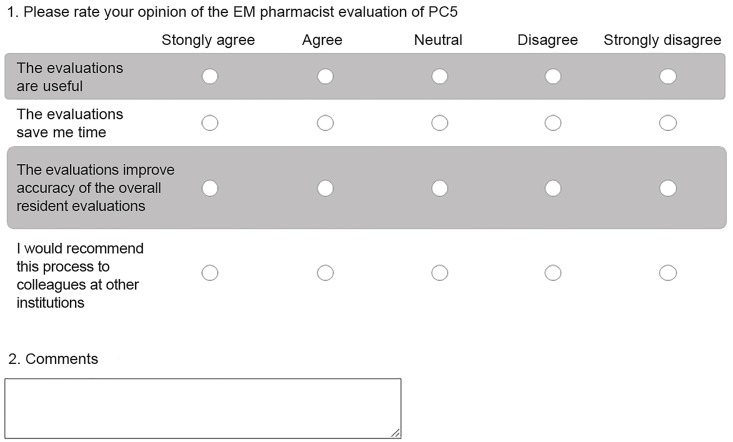
An anonymous survey instrument used to gather opinions of clinical competency committee members about the evaluation of emergency medicine residents by clinical pharmacists. *EM*, emergency medicine.

The pharmacists presented this report to the CCC, which was used with other evaluations to assess the PC5 sub-competency. Additionally, the pharmacists provided a written narrative that included observed patient and nursing interactions. These comments were available for incorporation into other competency evaluations such as professional values (PROF1), patient-centered communications (ICS1), and team management (ICS2). An anonymous survey of the CCC members was conducted using www.surveymonkey.com (SurveyMonkey Inc., San Mateo, CA) to elicit their feedback on the effectiveness of the pharmacists’ assessment (Figure 1).

## RESULTS

**Figure 2:**
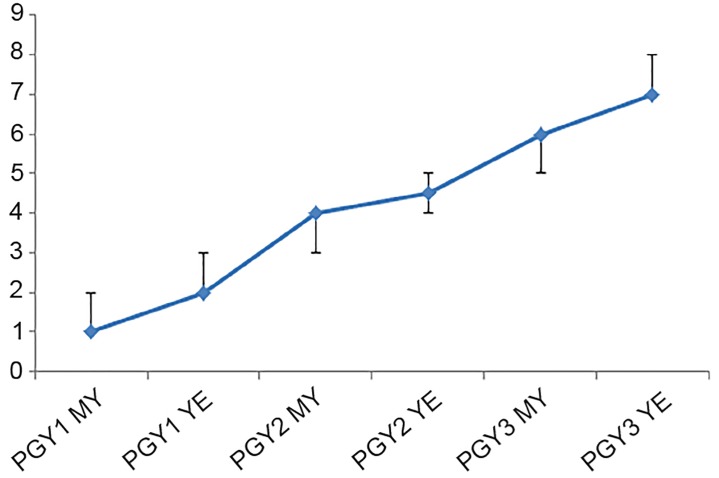
The median pharmacotherapy milestone scores assigned to residents by clinical pharmacists during each training year, at the mid-year (MY) and year-end (YE) evaluations. Interquartile range is represented by the bars. *PGY,* postgraduate year; *PC5*, pharmacotherapy sub-competency.

**Figure 3:**
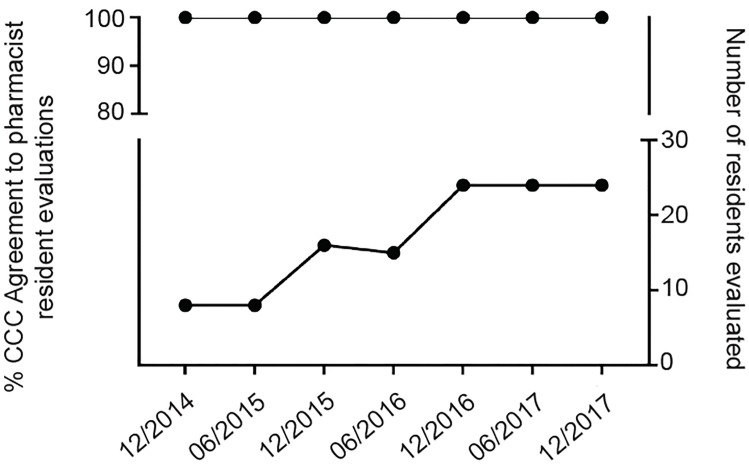
Members of the clinical competency committee agreed with the clinical pharmacists’ evaluation (top) of residents’ mastery of the pharmacotherapy sub-competency milestone, even as the number of residents increased (bottom). *CCC*, clinical competency committee.

Since introduction of pharmacist assessment of resident pharmacotherapy knowledge, seven biannual CCC meetings have occurred with 120 separate resident assessments made by the ED pharmacists and the CCC with regard to the PC5 sub-competency (Table 1). The range of PC5 scores improved over time for all classes evaluated (Figure 2). In all 120 assessments, there was 100% agreement between the pharmacist sub-competency recommendation and the CCC final assessment of the trainee over the 3.5-year period (Figure 3).

All CCC members responded to the survey except for the PD, who was ineligible due to input in the development of the survey instrument. All respondents agreed or strongly agreed that pharmacist evaluations were useful, saved time, and improved the accuracy of the overall resident evaluations (Table 2). Most respondents agreed or strongly agreed that they would recommend this process to other institutions.

## DISCUSSION

Using ACGME milestones is a step toward reliable assessment of a resident’s competence; however, these assessments can be problematic, particularly with regard to consistency and agreement of assessment level.^5^,^6^ The pharmacists who evaluate our residents’ pharmacotherapy competency have four years of doctoral training in pharmacology and therapeutics (In contrast, only one to two blocks of pharmacology are included in most medical school curriculums prior to clinical rotations). Additionally, pharmacy residencies often include preceptor development, including techniques for completing evaluations and providing feedback both during initial residency training and as ongoing education for accredited preceptors. The use of ED pharmacists to assist in the assessment process of resident milestones, especially with the pharmacotherapy sub-competency, allows for resident learning opportunities from a non-physician clinical perspective, and also provides important input into the assessment of PC5. Given the complexities of this milestone, resident assessment in the real-world setting during actual patient care likely adds to the accuracy of the evaluation.

In our experience, the assessments were timely and well received, leading to the CCC’s 100% acceptance of the PC5 sub-competency assessments submitted by the pharmacists. They provide a different perspective beyond that offered by physician assessment and medical knowledge testing. Given their education and post-doctoral training leading to EM specialization and their board certification in pharmacotherapy, as well as their years of clinical experience working in the ED, we believe that pharmacists play a vital role in our multidisciplinary approach to evaluating resident milestones and that their input leads to better trained emergency physicians.

In August 2017, the American College of Medical Toxicology (ACMT) released a position statement in full support of having clinical pharmacists in the ED 24 hours a day.^7^ The ACMT statement came concurred with that of the 2015 American College of Emergency Physicians statement in support of clinical pharmacy services in the ED.^8^ Both statements referenced not only the safety aspects of having pharmacists in the ED – by reducing medication errors, tailoring patients’ therapy based on concurrent disease states, medications, allergies, and presenting symptoms, and positively impacting time-critical diagnoses therapies – but also a financial benefit for cost avoidance and improved reimbursement rates.^7^,^8^

Notwithstanding the usual barriers to evaluation, which include difficulty evaluating residents who have been on off-service rotations for a majority of the evaluation period and evaluating pharmacotherapy knowledge for uncommon scenarios, the pharmacist’s daily observation of the EM resident adds another layer of assessment that might not otherwise be available. ED pharmacists are a valuable asset within the department on many levels, and the addition of their resident-assessment capability only further highlights the academic and clinical value for full-time ED-based pharmacists. Their evaluation scores were accepted 100% of the time by the CCC, and their input was considered valuable and clinically accurate. As a result, the pharmacists’ assessments and comments on the PC5 sub-competency were consistently included in the CCC’s official milestone update. The perceived added value of non-physician provider input for other milestones was decided upon by the committee, and the ED pharmacists were officially included as formal members of the CCC. This use of a pharmacist as a resident assessor could be expanded across other specialties that use department-specific pharmacists, such as the intensive care unit setting, pediatrics, and internal medicine.

## LIMITATIONS

Our CCC members were not blinded to the pharmacist evaluators. They and the pharmacists know and work with each other on a regular basis. This could have influenced the survey results, perhaps by inflating the positive impressions of the pharmacists. In addition, the evaluative tool for assessing residents during the oral boards was not specifically validated for PC5. Lastly, the medication choices to assess the residents’ knowledge base were chosen by group consensus of the residency leadership and the pharmacists without validation specifically to the PC5. The pharmacists at our institution have significant training and experience in both EM and preceptorship, making them ideally suited to evaluate PC5 and participate in the CCC. Incorporating pharmacists without this level of training and experience could produce different results.

## CONCLUSION

Integrating ED-based clinical pharmacists into the assessment process of EM residents adds a valuable area of focus that may otherwise be difficult to obtain.

## Figures and Tables

**Table 1:** The pharmacist assessment tool of pharmacotherapy for use in mock oral boards.

	3 - Exemplary	2 - Competent	1 - Needs improvement
Pharmacotherapy selection	Identifies appropriate medication by name	Selects an effective medication in the correct class but does not select the optimum therapy	Unable to determine appropriate medication class
	Selected medication is effective for patient presentation	Selected medication is effective for patient presentation	Selected medication is not effective for patient presentation
	Selected medication considers all contradictions and warnings	Selected medication considers contraindications and major warnings	Selected medication is contraindicated or has major warnings not addressed during case
Dosing/route/frequency	Provides a complete pharmacotherapy recommendation including appropriate dose, route, and frequency	Provides a dose and route but does not verbalize frequency	Does not verbalize a dose or gives an incorrect dose, route, or frequency
		Refers to hospital policy or protocols to determine dose/route/frequency (e.g., “consult pharmacy”)	
Follow-up and monitoring	Has a strategy to proactively assess patient response to therapy	Does not have a proactive plan for monitoring response therapy	Does not assess patient’s response to therapy
	Able to identify common monitoring parameters	Able to identify common monitoring parameters	Not able to identify common monitoring parameters

**Table 2:** Rating of the clinical pharmacists’ assessment by the clinical competency committee.

	Strongly agree	Agree	Neutral	Disagree	Strongly disagree
Evaluations are useful	71.4 %	28.6%	0%	0%	0%
Evaluations save me time	85.7%	14.3%	0%	0%	0%
The evaluations improve accuracy of the overall resident evaluations	71.4%	28.6%	0%	0%	0%
I would recommend this process to colleagues at other institutions	71.4%	14.3%	14.3%	0%	0%

## References

[1] Nasca TJ, Philibert I, Brigham T (2012). The next GME accreditation system--rationale and benefits. N Engl J Med.

[2] Carter WA (2014). Milestone myths and misperceptions. J Grad Med Educ.

[3] Beeson MS, Carter WA, Christopher TA (2013). The development of the emergency medicine milestones. Acad Emerg Med.

[4] (2015). The Accreditation Council for Graduate Medical Education and the American Board of Emergency Medicine. The Emergency Medicine Milestone Project Secondary The Emergency Medicine Milestone Project.

[5] Goldflam K, Bod J, Della-Giustina D (2015). Emergency medicine residents consistently rate themselves higher than attending assessments on ACGME milestones. West J Emerg Med.

[6] Weizberg M, Bond MC, Cassara M (2015). Have first-year emergency medicine residents achieved Level 1 on care-based milestones?. J Grad Med Educ.

[7] Amercian College of Medical Toxicology (2017). The Role of Clinical Pharmacists in the Emergency Department. ACMT Position Statement.

[8] American College of Emergency Physicians (2015). Clinical Pharmacist Services in the Emergency Department. Ann Emerg Med.

